# ﻿Larval polyphagy of *Cataspilatesmarceloi* (Lepidoptera, Geometridae), a Neotropical geometrid moth with flightless females

**DOI:** 10.3897/zookeys.1186.112397

**Published:** 2023-12-19

**Authors:** Héctor A. Vargas

**Affiliations:** 1 Departamento de Recursos Ambientales, Facultad de Ciencias Agronómicas, Universidad de Tarapacá, Casilla 6-D, Arica, Chile Universidad de Tarapacá Arica Chile

**Keywords:** Andes, Asteraceae, brachyptery, polyphagous caterpillar, Solanaceae

## Abstract

Surveys in the arid shrubland of the central Andes revealed larval polyphagy for *Cataspilatesmarceloi* Vargas, 2022 (Lepidoptera, Geometridae, Ennominae, Boarmiini), a geometrid moth with flightless females. This discovery suggests that, as well as in the Holarctic fauna, larval polyphagy would have been important for the evolution of flightlessness among Neotropical geometrid moths of the tribe Boarmiini.

## ﻿Introduction

The image that usually comes to mind when thinking about Lepidoptera is one of a winged insect. However, wing reduction has evolved independently in many lineages of this insect order (Heppner 1991; Sattler 1991). Cases of wing reduction in the highly diverse moth family Geometridae are restricted to females, and involve species of different subfamilies (Sattler 1991). A molecular phylogenetic study of Holarctic geometrid moths of the subfamily Ennominae revealed seven independent transitions to wing reduction, four of which occurred in the tribe Boarmiini (Wahlberg et al. 2010). Divergence between the oldest clade in this tribe with flightless females and its winged sister group was dated to about 37 Ma, and only 7 Ma for the youngest transition (Murillo-Ramos et al. 2021).

Wing reduction remained overlooked in the Neotropical fauna of Boarmiini until the recent discovery of brachypterous females of *Cataspilatesmarceloi* Vargas, 2022 on the arid western slope of the central Andes of northern Chile. Its genitalia morphology suggests closeness to *Glena* Hulst, 1896, *Glenoides* McDunnough, 1922 and some species currently included in *Physocleora* Warren, 1897, all of which have winged females (McDunnough 1920; Pitkin 2002). Furthermore, an analysis based on mitochondrial DNA sequences clustered *C.marceloi* with “*Physocleora*” sp. (BOLD Process ID GEOCO032-20, Sample ID LMR_Geo035) (Vargas 2022). Recent phylogenetic studies revealed polyphyly for *Physocleora* and clustered its species near *Glena*, *Glenoides* and other genera distantly related to the Holarctic lineages of Boarmiini with flightless females (Brehm et al. 2019; Murillo-Ramos et al. 2021). Although further studies are needed to understand the phylogenetic relationships of *C.marceloi*, the currently available data suggest that transition to wing reduction in this Neotropical geometrid moth would have been independent of those previously recognized in the Holarctic Boarmiini.

In addition to robust phylogenies, knowledge of natural history of geometrid moths provides important insights to improve the understanding of their evolutionary patterns. Based on the integration of these two aspects for the tribe Operophterini of the subfamily Larentiinae, Snäll et al. (2007) proposed that some groups are predisposed to the evolution of wing reduction due to certain previously evolved permissive life history traits, a hypothesis consistent with results of subsequent studies dealing with Holarctic Ennominae (Wahlberg et al. 2010). A broad host plant range is an especially relevant permissive trait (Snäll et al. 2007; Wahlberg et al. 2010). Larval polyphagy is associated with the four independent transitions to wing reduction in the Holarctic Boarmiini (Wahlberg et al. 2010). In contrast, a single host plant (*Adesmiaspinosissima* Meyen, Fabaceae) has been documented for the South American *C.marceloi*, hindering the understanding of this case of flightlessness. The aim of this contribution is to provide new host records that reveal larval polyphagy for this little-known geometrid moth.

## ﻿Materials and methods

Larvae were collected using a beating sheet on the shrubs *Baccharistola* Phil. (Asteraceae) and *Fabianaramulosa* (Wedd.) Hunz. & Barboza (Solanaceae) in April, 2023 near Socoroma Village (18°16'42"S, 69°34'15"W), Parinacota Province, at 3400 m elevation on the western slope of the Andes of northern Chile. This is the same sampling site of a paratype of *C.marceloi*, about 10 km northwest of the type locality. The larvae collected were brought to the laboratory in plastic vials with parts of the respective plant and reared until they finished feeding and pupated. The abdomen of each emerged adult was removed and placed in hot KOH 10% for a few minutes for dissection of the genitalia, which were stained with Eosin Y and Chlorazol Black and mounted on slides with Euparal. The specimens studied and their genitalia slides are deposited in the “Colección Entomológica de la Universidad de Tarapacá” (IDEA), Arica, Chile.

Genomic DNA was extracted from legs of two specimens using the QIAamp Fast DNA Tissue Kit (Qiagen). PCR amplification of the barcode region (Hebert et al. 2003) was performed with the primers LCO1490 and HCO2198 (Folmer et al. 1994) using a program of 5 min at 94 °C, 35 cycles of 30 s at 94 °C, 30 s at 47 °C, 1 min at 72 °C and a final elongation step of 10 min at 72 °C. DNA purification and sequencing were performed at Macrogen Inc. (Santiago, Chile). Additional sequences of *C.marceloi* were downloaded from BOLD (Ratnasingham and Hebert 2007). Sequence alignment with the ClustalW method and assessment of the genetic distances with the Kimura 2-Parameter (K2P) method were performed in the software MEGA11 (Tamura et al. 2021).

## ﻿Results

**Figures 1–5.**
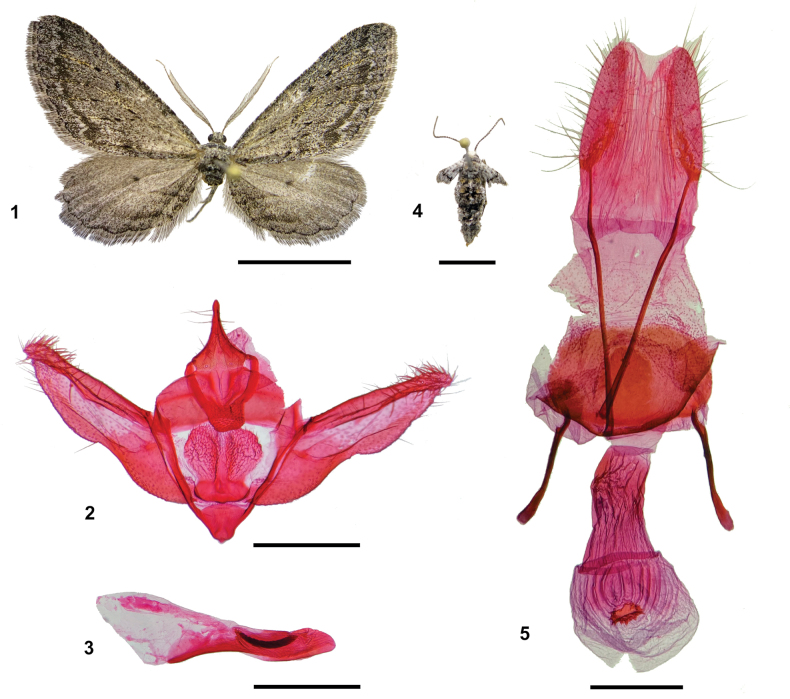
Adults of *Cataspilatesmarceloi* reared from larvae collected on two new host plants **1** male, abdomen removed, dorsal view, reared from *Baccharistola* Phil. (Asteraceae) **2** male genitalia, phallus removed, ventral view **3** phallus, lateral view **4** female, dorsal view, reared from *Fabianaramulosa* (Wedd.) Hunz. & Barboza (Solanaceae) **5** female genitalia, ventral view. Scale bars: 10 mm (**1**); 0.5 mm (**2, 3, 5**); 5 mm (**4**).

One male and two females were reared from larvae collected on *B.tola*, and one male and seven females from *F.ramulosa*. The emerged adults were identified as *C.marceloi* based on morphology (Figs 1–5).

Two identical DNA barcodes were obtained from the male reared from *B.tola* (BOLD Process ID CATMA003-23) and one female from *F.ramulosa* (BOLD Process ID CATMA004-23). The genetic divergence of these two specimens with the holotype (BOLD Process ID CATMA001-22) and one paratype (BOLD Process ID CATMA002-22) of *C.marceloi* was 1.4–1.5% (K2P). As indicated in the original description, the holotype and paratype of *C.marceloi* were reared from larvae collected on the shrub *A.spinosissima* near Murmuntani. The sequences from Socoroma and Murmuntani were assigned to different BINs (BOLD:AFL2832 and BOLD:AEW3146, respectively) with a genetic divergence of 1.4% (K2P) between the two clusters.

Although DNA barcode analysis is extremely useful for taxonomic identifications, different BINs not always correspond to different species (Prieto et al. 2021; Meier et al. 2022). A few cases of interspecific DNA barcode divergence similar to that found between the two BINs of *C.marceloi* have been described for geometrid moths (e.g. Wanke et al. 2019). However, genetic divergence between congeneric species of this moth family is generally deeper (e.g. Hausmann et al. 2009; Hausmann and Huemer 2011; Wanke et al. 2020). Accordingly, the two BINs recognized by BOLD for *C.marceloi* are here considered as conspecific, as no additional differences were found between the specimens from Murmuntani (type locality) and Socoroma. The genetic divergence between specimens from the two localities could be due to the low dispersal capacity of the flightlessness females.

### ﻿Material examined

CHILE • 1 ♂; Parinacota, Socoroma; May 2023; H.A. Vargas leg.; ex-larva; *Baccharistola*; April 2023; HAV-1666 [genitalia slide]; CATMA003-23 [BOLD Process ID] • 1 ♀; same collection data as for preceding; HAV-1667 [genitalia slide] • 1 ♀; same collection data as for preceding • 1 ♂; same collection data as for preceding; *Fabianaramulosa*; April 2023; HAV-1679 [genitalia slide] • 1 ♀; same collection data as for preceding; HAV-1674 [genitalia slide]; CATMA004-23 [BOLD Process ID] • 6 ♀♀; same collection data as for preceding; all in IDEA.

## ﻿Discussion

The results reveal that the previous record of a single host underestimated the host range of *C.marceloi*. The discovery of its larvae feeding on shrubs belonging to two additional families (Asteraceae and Solanaceae) is a clear demonstration of polyphagy for this little-known Neotropical geometrid moth.

Agreeing with the hypothesis of Snäll et al. (2007) for evolution of flightlessness in Operophterini, Wahlberg et al. (2010) proposed that the winged ancestor of lineages with flightless females among the Boarmiini of the Holarctic forests would have been a slow flying moth with polyphagous larvae that used deciduous trees as hosts. The discovery of larval polyphagy in *C.marceloi* suggests that this attribute might also have been important in the transition to flightlessness among Neotropical Boarmiini. Together with larval polyphagy, Wahlberg et al. (2010) mentioned spring larval feeding, overwintering as egg or pupa, and adults flying late/early season as permissive traits for evolution of wing reduction among Holarctic Boarmiini. All these traits would have reduced the importance of female flight, facilitating the transition from winged to wingless females (Snäll et al. 2007). The last three attributes would have allowed the phenology to adjust to the food availability in the Holarctic forests. In contrast, larvae of *C.marceloi* were collected in autumn, adults reared from these larvae emerged in the same season, and it remains unknown whether it has any overwintering stage, suggesting that the set of permissive traits for evolution of flightlessness among Neotropical Boarmiini could be at least partially different from that recognized for the Boarmiini of Holarctic forests.

**Figure 6.**
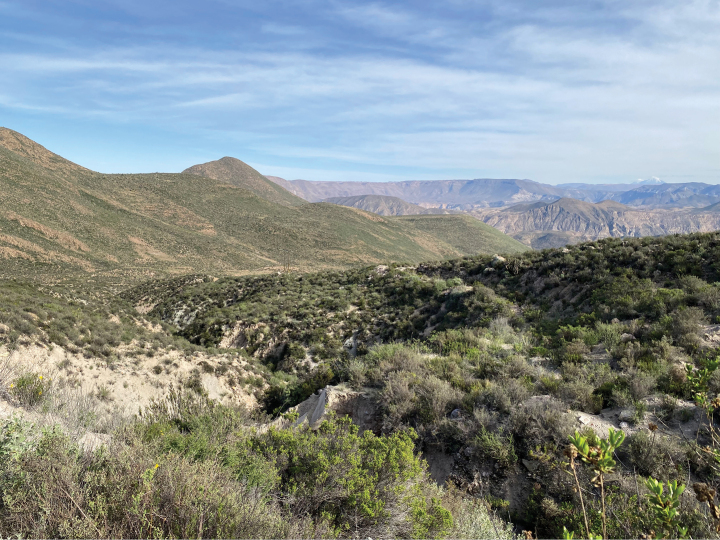
Sampling site of larvae of *Cataspilatesmarceloi*. A shrubland at 3400 m elevation on the arid western slope of the central Andes, in the surroundings of Socoroma Village, Parinacota Province, northern Chile.

The ecological context of the area inhabited by *C.marceloi* (Fig. 6) differs drastically from that of the Holarctic forests. The high elevation shrubland of the arid western slope of the central Andes has a tropical xeric climate with most rain concentrated in summer (Luebert and Pliscoff 2006), which provides water input for fast development of a highly seasonal vegetation cover (Muñoz and Bonacic 2006). The phenology of geometrid moths has been little explored in this area, but the available information suggests that different patterns coexist. Two cases of pupal dormancy have been recorded, in one of which larval feeding appears to be synchronized with highest vegetation cover (Vargas 2021), while larval feeding and adult emergence throughout the year has been recorded for two species (Vargas et al. 2020, 2022). Although the highest level of vegetation cover typically occurs shortly after summer rains, some shrubs of this area, including the hosts of *C.marceloi*, maintain some growth during the dry season (Muñoz and Bonacic 2006), providing food substrate for associated phytophagous Lepidoptera. Thus, a strict adjust in the phenology of a given life stage to a narrow period of the year might have been unnecessary for evolution of wing reduction in *C.marceloi*.

Scientific interest in the South American fauna of Geometridae has increased during the last 20 years, improving the understanding of biodiversity patterns and evolutionary relationships (e.g. Brehm 2002; Zamora-Manzur et al. 2011; Brehm et al. 2016, 2019; Ramos-González et al. 2019; Moraes et al. 2021; Murillo-Ramos et al. 2021; Machado et al. 2022). Further studies on the natural history and phylogeny of *C.marceloi* and close relatives are encouraged to disentangle the evolutionary history of wing reduction among Neotropical geometrid moths of the tribe Boarmiini.

## ﻿Additional information

### Author ORCIDs

Héctor A. Vargas https://orcid.org/0000-0002-5355-3157

### Data availability

All of the data that support the findings of this study are available in the main text.
